# Characterization of Porous Structures of Cellulose Nanofibrils Loaded with Salicylic Acid

**DOI:** 10.3390/polym12112538

**Published:** 2020-10-30

**Authors:** Birgitte Hjelmeland McDonagh, Gary Chinga-Carrasco

**Affiliations:** 1 SINTEF Industry, P.O. Box 4760 Torgarden, 7034 Trondheim, Norway; birgitte.mcdonagh@sintef.no; 2 RISE PFI, Høgskoleringen 6b, 7491 Trondheim, Norway

**Keywords:** nanocellulose, scaffolds, chemical elicitor, plant resistance, pesticide

## Abstract

Bleached and unbleached pulp fibers were treated with 2,2,6,6-tetramethylpyperidine-1-oxyl (TEMPO) mediated oxidation to obtain cellulose nanofibrils (CNFs). The resulting bleached and unbleached CNFs were mixed with salicylic acid (0, 5, 10, 20 wt%) before casting and freeze-drying or 3D-printing. A series of methods were tested and implemented to characterize the CNF materials and the porous structures loaded with salicylic acid. The CNFs were characterized with atomic force microscopy and laser profilometry, and release of salicylic acid was quantified with UV-visible absorbance spectroscopy, conductivity measurements, and inductive coupled plasma mass spectrometry (ICP-MS). Fourier-transform infrared spectroscopy (FTIR) complemented the analyses. Herein, we show that aerogels of bleached CNFs yield a greater release of salicylic acid, compared to CNF obtained from unbleached pulp. The results suggest that biodegradable constructs of CNFs can be loaded with a plant hormone that is released slowly over time, which may find uses in small scale agricultural applications and for the private home market.

## 1. Introduction

Defending crops against airborne pathogens and insects is a multimillion dollar industry that has severe impacts on the environment and human health [1]. The major reason for its environmental impact is use of non-specific chemicals that are toxic to a range of microorganisms as well as terrestrial and aquatic animals [2]. It is of imperative importance to develop green pesticides that provide healthy and nutritious crops that at the same time reduce the environmental impact of industrialized agriculture. One strategy to solve this problem could be to take advantage of the plants’ own defense system and exploit existing signaling pathways to strengthen the plants’ resistance to pathogens and abiotic stresses.

Plants are protected against attack from phytopathogens and fungi mainly through bark, waxy cuticle layers, and cell walls. Other exogeneous factors such as UV-radiation, salinity, extreme temperatures, and drought also have an impact on the plants’ ability to grow and bear fruiting bodies [3]. In addition to structural defense mechanisms, plants can trigger an inducible immune system when exposed to external threats. The strongest immune response is effector-triggered immunity (ETI). Molecules involved in ETI can be referred to as phytohormones, and include salicylic acid, ethylene, and jasmonic acid [4]. In particular, salicylic acid plays a critical role in a parallel resistance pathway called systemic acquired resistance (SAR). This pathway is non-specific and long-lasting, and protects the plant against a broad range of microorganisms [5]. This means that when a plant is infected by a pathogen, the SAR response ensures that non-infected and distal plant tissue is warned about the incoming threat and causes upregulation of a large number of pathogenesis-related genes [6].

Salicylic acid can be administered exogenously through the rooting system via the soil medium. Salicylic acid is taken up by plants through roots, and has been shown to alleviate salt stress and promote growth both under saline and non-saline conditions [7,8]. Salicylic acid has also been shown to promote flowering [9,10], as well as protect plants from toxic effects of insecticides [11], and reduce uptake of toxic ions [12,13]. This means that salicylic acid can act as a plant defense chemical when administered through the soil medium. However, salicylic acid is not used today as an alternative pesticide, most likely because of its cost and inability to absorb across cuticle layers. Because of its cost, salicylic acid must be administered locally and preferably directly to the soil with a system that can release salicylic acid over time. This requires a porous vehicle that can easily be loaded with salicylic acid and release it once in contact with soil. Methods to characterize release of salicylic acid are thus required and this is part of the motivation of the present study.

Cellulose nanofibrils (CNFs) can be applied for forming porous vehicles for salicylic acid. CNFs are obtained from lignocellulosic biomass and is a biodegradable material [14,15]. Various studies have demonstrated that CNFs have diameters of less than 100 nm and lengths in the micrometer-scale [16,17,18,19,20]. Due to their particular characteristics, CNFs can create stable and highly porous structures that can be embedded with drugs or chemicals through chemical modification, purification, and adequate manufacturing [21,22]. CNFs therefore offer a unique platform as a soil degradable material with drug delivery abilities.

The aim of this study was to characterize two different TEMPO CNFs, from unbleached and bleached kraft pulp fibers and describe how the CNFs can be applied to form three-dimensional porous structures to release salicylic acid as a model elicitor molecule. Unbleached pulp was used as comparison to bleached pulp, as unbleached pulp requires less processing steps during production, the pulp fibers are thus cheaper than bleached pulp fibers, and most probably would have better performance in life cycle assessments, which is also environmentally beneficial. One of the specific goals of the study was to develop characterization methods to quantify release of salicylic acid that will be applied in ongoing and future plant growth trials.

## 2. Materials and Methods

### 2.1. Materials

Salicylic acid (ACS reagent ≥ 99.0%, CAS: 247588-500g) was purchased from Sigma Aldrich (Saint-Louis, Missouri, MI, USA). The raw material for production of CNFs was bleached and unbleached Pinus radiata pulp fibers provided by CMPC (Nacimiento, Chile). The content of cellulose, hemicellulose, and lignin of the unbleached pulp was 80.9, 12.7, and 6.4%, respectively. The content of cellulose, hemicellulose, and lignin of the bleached pulp was 87, 12.2, and 0.8%, respectively [23].

### 2.2. Production and Characterization of CNFs

The pulp fibers (bleached and unbleached) were treated with TEMPO-mediated oxidation, using 6 mmol sodium hypochlorite (NaClO, concentration 9%)/g dry matter content cellulose. TEMPO and NaBr were dissolved in water and added to the pulp. NaClO was added and dropwise addition of 0.5 M NaOH was used to maintain the pH at 10. The reaction time was approx. 60 min, until the pH was constant. For details see [24]. The pre-treated fibers were homogenized with an Ultra-turrax equipment (IKA^®^-Werke GmbH & Co. KG, Staufen, Germany) for 6 min at 24,000 rpm. The concentration of the CNFs dispersion was 2 wt%. The CNFs from the bleached pulp are hereafter referred to as bleached CNFs, while the ones based on unbleached pulp are referred to as unbleached CNFs.

Conductometric titration was applied to quantify the carboxylic acid content, following the procedure described by Saito and Isogai [24]. An automatic titrator was applied (Metrohm 902 Titrando, Evenwood, UK) and the carboxyl groups content was estimated from the titration curve.

Laser profilometry (LP, IVT-Lehmann Messtechnikk AG, BielBiene, Switzerland) of CNF films was performed to assess the fibrillation degree. CNF dispersions of 0.2 wt% were casted in Petri dishes to form films (20 g/m^2^) and allowed to dry for 5–7 days at ambient conditions. Samples (20 mm × 10 mm) were mounted on objective microscopy slides with double-sided tapes and coated with a layer of gold. Ten images (1000 µm × 1000 µm, resolution 1 µm/px) from each sample (unbleached and bleached CNFs films) were acquired with the Laser Profilometry system. The images were processed with the ImageJ program (version 1.53b, National Institute of Health, Kansas, MI, USA) and the surface roughness at various lateral wavelengths was quantified with the SurfCharJ plugin (version 1q, Trondheim, Norway).

The nano-morphology of the CNFs was analyzed with a Veeco multimode V (Bruker Instruments, Billerica, MA, USA) at room temperature.Atomic force Microscopy (AFM) tips (SA-air) with a spring constant ~0.4 N m^−1^ were purchased from Bruker AFM (Billerica, MA, USA). Freshly cleaved mica was attached to a magnetic stub with double-sided tape. Top layers of mica were then removed before sample application. Twenty-five microliters of diluted CNFs (MQ-water, 0.5 wt%) were applied to mica and dried at room temperature overnight before analysis. Height and peak force error data were collected at a resolution of 1024 samples/line with an aspect ratio equal to 1.

### 2.3. Characterization of CNFs Loaded with Salicylic Acid

Loading of salicylic acid was performed by mixing salicylic acid (0, 5, 10, 20 wt%) directly with the CNFs hydrogel. The CNFs-salicylic acid mixtures (0.3 g) were added to 96-well plates with three replicates for each salicylic acid concentration. Samples were then frozen (−20 °C) and lyophilized (freeze-drying). Samples were weighed after freeze-drying before measuring release of salicylic acid in MilliQ-water.

Fourier-transform infrared spectroscopy (FTIR) was conducted on freeze-dried samples to assess the unbleached and bleached CNFs and loaded with 20 wt% salicylic acid. The FTIR was a Frontier, FTIR Spectrometer fitted with a universal ATR (UATR) sampling accessory, by Perkin Elmer (Waltham, MA, USA). The infrared spectra were collected in the range of 800–4000 cm^−1^.

Unbleached and bleached CNFs with and without salicylic acid (10 wt%) were printed with a Regemat3D printing unit to test the forming of porous constructs with controlled 3D structure. The constructs consisted of 4 layers with a target height of 2 mm. The lateral dimensions were 20 × 40 mm.

The freeze-dried structures (3D printed or casted) were sputtered with a layer of gold and assessed with SEM (Hitachi scanning electron microscope, SU3500, Hitachi High-Tech Corporation, Tokyo, Japan), in secondary electron imaging mode, using an acceleration voltage of 5 kV and working distance of 5–10 mm. Samples were cut, and mounted with the lateral side facing upwards, to show internal porosity.

### 2.4. Quantification of Release of Salicylic Acid

A time-dependent release study was conducted by adding CNFs with salicylic acid to MilliQ-water (0.2 mg CNFs/mL) and measuring the conductivity as a function of time for 24 h. A standard curve of salicylic acid was prepared for UV-vis spectroscopy (λabs = 297 nm) with a Shimadzu UV 1800 (Shimadzu Schweiz GmbH, Reinach BL, Switzerland). At the end-point (t = 24 h) the supernatant was measured with UV-vis spectroscopy at λabs = 297 nm and the amount of salicylic acid released after 24 h was calculated based on the standard curve.

Endpoint samples were also analyzed for sodium content with Inductive Coupled plasma-mass spectrometry (ICP-MS) to subtract sodium contribution from conductivity measurements. The ICP-MS instrument was a MS Triple Quad, Agilent-8800, by Agilent (Santa Clara, CA, USA).

## 3. Results and Discussion

### 3.1. Characterization of Bleached and Unbleached CNFs

TEMPO-mediated oxidation is used to oxidize the C6 hydroxylic groups to carboxylic acid moieties on the cellulose backbone. TEMPO-mediated oxidation is more efficient on bleached cellulose [25], as lignin reduces the reaction time and thus the oxidation levels [26]. After TEMPO-mediated oxidation the cellulose nanofibrils will have a higher carboxyl group content, compared to nanofibrils that have been produced without chemical pretreatment [27]. The carboxylic acids are negatively charged at pH above the pKa, i.e., the charge density increases, causing electrostatic repulsion between nanofibrils and this increases the degree of fibrillation [28]. A cellulose sample with more lignin residues would impair TEMPO-oxidation efficiency and reduce charge density. As shown in Table 1, bleached CNFs showed a higher carboxyl group content (average charge) than unbleached CNFs, and this is most likely an effect of increased lignin content in the latter [23]. With a less efficient TEMPO-oxidation the overall charge density and electrostatic repulsion is smaller which in turn is likely to affect the degree of fibrillation.

**Figure 1:**
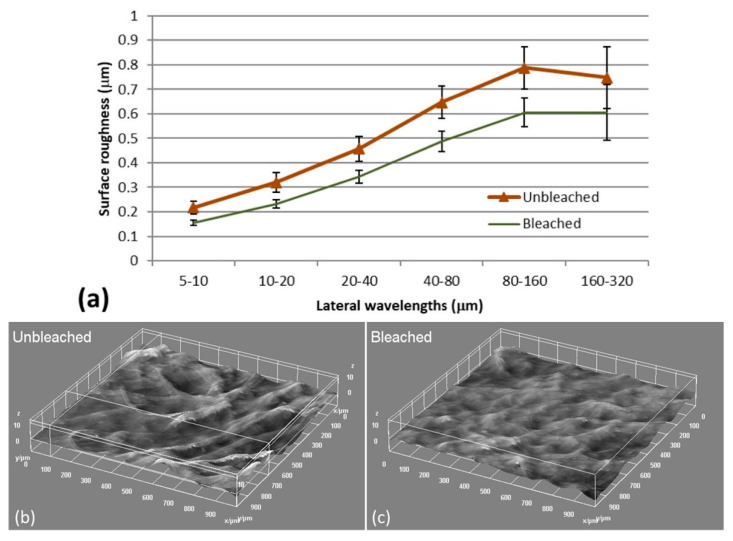
(**a**) Surface roughness analysis on films prepared from bleached and unbleached CNFs showed that the latter had a higher surface roughness than the former. The roughness values are given with the corresponding standard deviation, *n* = 10. Representative laser profilometry images of unbleached (**b**) and bleached samples (**c**) show how residual fibers contribute to the roughness for the CNFs film.

Laser profilometry has previously been applied to quantify the degree of fibrillation of CNFs materials, represented by an estimation of the amount of residual fibers [29]. A rougher surface of air-dried CNFs films means that there are a larger fraction of residual fibers and thus less fraction of cellulose nanofibrils [29]. Laser profilometry images and roughness analysis (Figure 1) show that unbleached CNFs have a rougher surface than bleached CNFs which indicates a lower degree of fibrillation and confirms a larger fraction of residual fibers.

**Figure 2:**
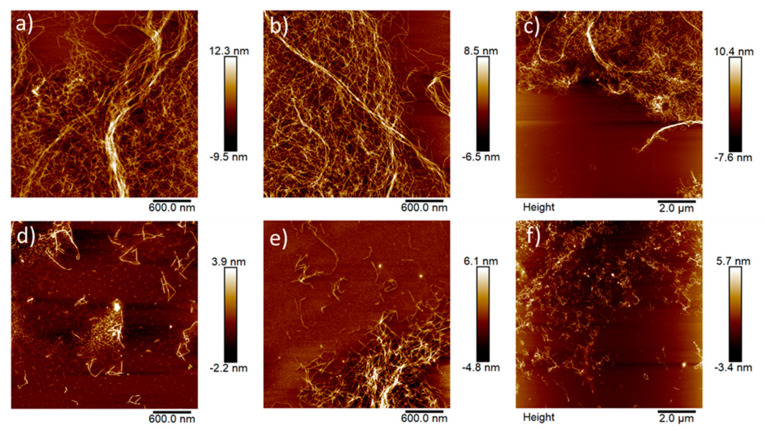
AFM of unbleached ((**a**–**c**) less fibrillated) and bleached ((**d**–**f**) more fibrillated) CNFs.

To further investigate the morphology of the nanofibrillated materials, the CNFs were characterized with AFM. AFM images (Figure 2) show a clear difference between the two CNFs qualities. The AFM images of bleached CNFs confirmed less occurrence of residual fibers compared to the unbleached CNFs. Consequently, bleached CNFs had a higher occurrence of nanofibrils, which is most likely an effect of higher surface charge and increased electrostatic repulsion (Figure 2).

### 3.2. Release Studies of Salicylic Acid from CNF Aerogels

**Figure 3:**
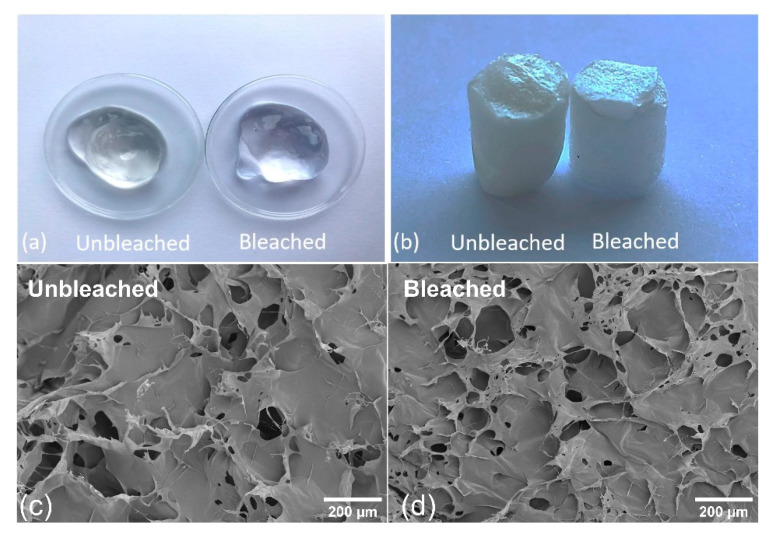
Images of bleached and unbleached CNFs hydrogels (**a**) and freeze-dried aerogels (**b**). The freeze-dried aerogel samples were approx. 5 mm in diameter and 10 mm in height. Note the slightly different yellow color caused by the lignin content in the unbleached CNF sample. SEM images of the unbleached (**c**) and bleached (**d**) freeze-dried aerogels. The SEM images were acquired at 100× magnification.

The CNF gels and aerogels from the two pulps (bleached and unbleached) are given in Figure 3a,b. TEMPO-mediated oxidation introduces functional groups to cellulose. The pKa,COOH for salicylic acid is 2.97, while the pKa of C6-groups on cellulose (uronic acid) after TEMPO-oxidation is 2.8–3.7 [30]. This means that at pH values of 4–5, both carboxylic groups on polyuronic acid and salicylic acid are negatively charged which leads to increased electrostatic repulsion and potentially increased release of salicylic acid. Compared to cellulose nanocrystals (CNC), CNFs have a higher flexibility and thus a greater tendency to entangle which makes the material more suitable for network formation [31]. The three-dimensional structure of CNFs can also be tailored based on the TEMPO-mediated oxidation and degree of protonation [32]. Representative SEM images of bleached and unbleached freeze-dried CNF structures are presented in Figure 3c,d, which exemplify the pore structure of the formed aerogels. Note the size of the pores ranging from roughly 50 to 200 mm. Such pores are formed due to the ice crystal formation during freezing.

**Figure 4:**
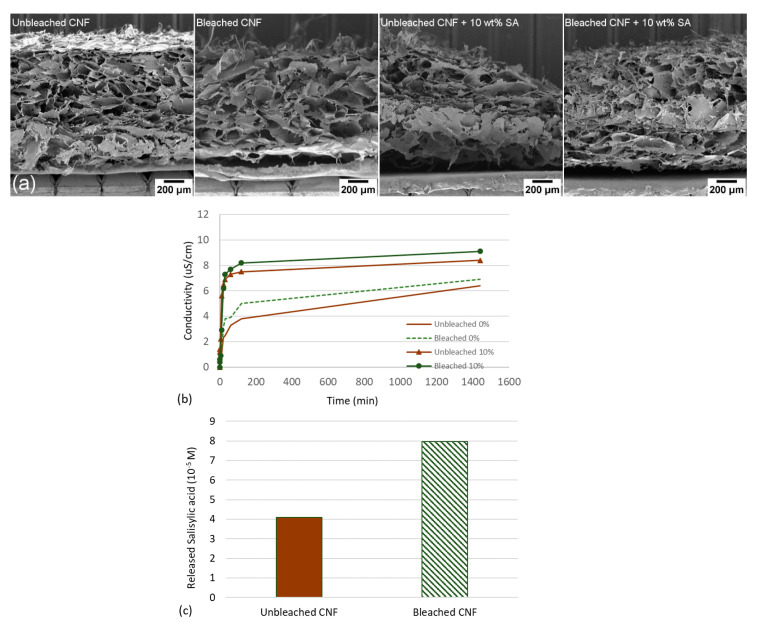
(**a**) SEM images of local regions of porous 3D printed constructs. Note the disrupted structure of the unbleached CNFs loaded with 10 wt% salicylic acid. (**b**) shows conductivity in supernatant as a function of time, while (**c**) shows release of salicylic acid in supernatant after 24 h.

The most common methods to prepare CNFs aerogels are freeze-drying or critical point drying. However, more control of the macroscopic geometry can potentially be achieved through 3D-printing, which offers the possibility to tailor 3D constructs by depositing the CNFs gels layer by layer in a pre-defined shape and with a given porosity. Pores with diameters of roughly 100–200 µm are typical for these types of freeze-dried material [33]. Hence, we explored 3D printing to prepare CNFs constructs with salicylic acid as an attempt to control the structure and porosity and its effect on the release profile. Specifically, bleached and unbleached CNFs were mixed with different weight ratios of salicylic acid (0, 10 and 20 wt%) before 3D-printing and freeze-drying. The 3D-printed scaffolds were characterized with SEM (Figure 4a). Note that the structures of the samples containing salicylic acid appeared to be more disrupted compared to the corresponding bleached and unbleached samples without salicylic acid (Figure 4a). This was due to the difficulties during 3D printing caused by the samples containing salicylic acid. It is however important to note that SEM images of CNFs self-assembled structures are performed on a dry material, which means that the structure is likely to be very different once dispersed in water. After characterization of the 3D-printed CNFs, the material’s ability to release salicylic acid was evaluated (Figure 4b).

Release profiles of salicylic acid from 3D printed CNFs show that bleached CNFs release more salicylic acid after 24 h compared to unbleached CNFs (Figure 4b). This may be an effect of the differences in surface charge of the nanofibrils (Table 1). Bleached CNFs has a carboxylic acid content of 1376 µmol/g, which is 30% higher than the carboxylic acid content of the unbleached sample. Our results show that 3D printed constructs of bleached CNFs released more salicylic acid than unbleached CNFs (Figure 4b,c).

It was difficult to 3D print constructs containing 10 wt% salicylic acid, especially for the unbleached sample. This is most probably due to the relatively large content of residual micrometer-sized fibers (Figure 2) which may block the nozzle. Hence, we put our efforts into testing aerogels that were casted and freeze-dried to assess the release profiles of salicylic acid at various ratios (0, 5, 10, 20 wt%).

**Figure 5:**
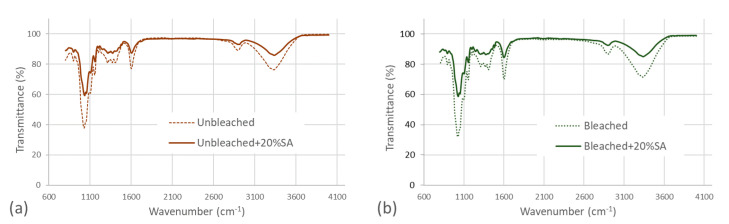
FTIR spectra of unbleached samples (**a**) and bleached samples (**b**).

An FTIR analysis was performed to assess the chemical modifications of the CNF and aerogels (without and with 20 wt% salicylic acid) (Figure 5). Only the samples with the highest content of salicylic acid were included to secure that the content could be detectable by the FTIR system. The spectra reveal the typical peaks at 3300 and 2900 cm^−1^ of cellulose materials, corresponding to the stretching vibration of the OH and CH groups. The bands between 1450 and 1250 cm^−1^ correspond to the CH or OH bending and CH stretch in hemicelluloses [34]. Note that there is no evident peak at 1510 cm^−1^ in the assessed CNF samples. This peak has been related to the C = C symmetrical stretching of aromatic rings that are typical of lignin [35]. This indicates that the lignin of the unbleached pulp (lignin content quantified to 6,4%) have been reduced considerably during the TEMPO-mediated oxidation and was not detected by FTIR. We have previously demonstrated that TEMPO mediated oxidation reduces the amount of lignin considerably, confirming these findings [36]. The peak at 1610 cm^−1^ is attributed to the C = O stretching vibration of carboxyl groups and is an indication of the carboxylation at the C6 position of the glucose unit of cellulose. The peak at 1610 cm^−1^ of the bleached CNF is more pronounced than the corresponding unbleached CNF and confirms the larger occurrence of carboxyl groups in bleached CNFs quantified by conductometric titration (Table 1). A clear difference is observed between the neat CNF samples and the samples loaded with salicylic acid, due to the reduction of CNF mass and thus an increase in transmittance. On the other side, a minor increase is detected in the peaks at 1700 cm^−1^, 2890 cm^−1^, and 2920 cm^−1^ that have been previously reported to appear in carboxymethyl cellulose loaded with salicylic acid [37].

**Figure 6:**
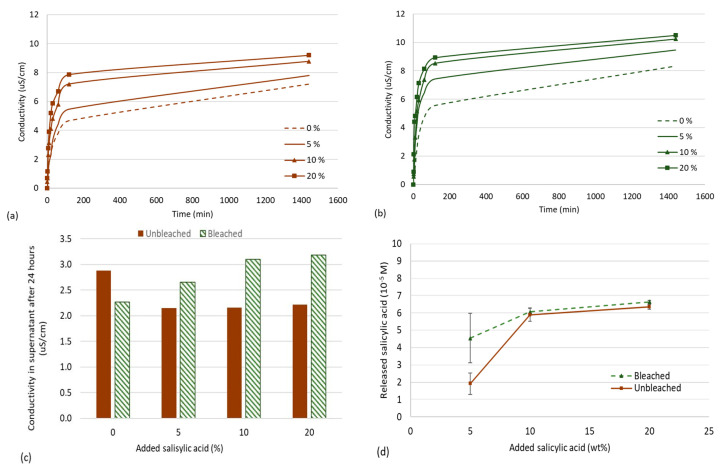
Conductivity measurements were performed over 24 h on (**a**) bleached CNFs and (**b**) unbleached CNFs loaded with 0, 5, 10, and 20 wt% salicylic acid. The supernatant was retrieved after 24 h, and (**c**) ICP-MS was conducted on all supernatants to subtract the contribution of sodium to the conductivity, assuming that 1 mg Na/L equals 2 µS/cm. A standard curve on salicylic acid had been prepared with UV-vis spectroscopy, and (**d**) the supernatant after 24 h was analyzed with UV-vis spectroscopy (λabs = 297 nm) and the corresponding concentration of salicylic acid in the supernatant was quantified. The average values in (**d**) are given with the corresponding standard deviation, *n* = 3.

The aerogel samples were immersed in distilled water (pH = 7.0, Conductivity = 0.9 µS/cm) and the conductivity was measured to further study the release profile as a function of time and salicylic acid loading. When the CNFs aerogel is immersed in water it will swell and release ionic species. This outward diffusion of ions is detected as an increase in conductivity. Conductivity is a non-specific measurement, as it is not possible to distinguish which ions that are diffusing from the matrix, but it is a method to determine how rapidly charged molecules/atoms are released. In the aerogels prepared here, we expect that salicylic acid, sodium, and cellulose nanofibrils contribute to conductivity. Salicylic acid is deprotonated (pKa, COOH = 2.97) at neutral pH and salicylic acid and counterions would therefore contribute to increased conductivity (Figure 6a,b). Although only one sample was measured for each timepoint, the conductivity profiles follow similar trends at reasonable levels of conductivity. We thus consider the results shown in Figure 6a,b to be reliable.

It is also likely that the increase in conductivity is caused by oxidized cellulose nanofibrils that are released from the CNFs matrix. To elucidate which ionic species were present in the supernatant, we analyzed the supernatant for sodium with inductive coupled plasma mass spectrometry (ICP-MS). The sodium content of the supernatant was found to be highest in the bleached CNFs. Sodium is a counter ion for the carboxylic groups after TEMPO-mediated oxidation. Sodium contribution was then subtracted from the conductivity measurements to give an indication of the concentration of other ions in the supernatant. All bleached CNFs loaded with salicylic acid had an overall higher conductivity compared to unbleached CNFs (Figure 6c), which is expected to be related to the higher surface charge of the bleached CNFs and the corresponding electrostatic repulsion with salicylic acid.

UV-visible spectroscopy was conducted to quantify salicylic acid released from the CNF aerogels (Figure 6d). The results show that salicylic acid is present in the supernatant, and that bleached CNFs releases more salicylic acid. This may be an effect of the higher charge density of bleached CNFs, which contributes to a larger swelling of CNFs aerogels in water and thus may have caused an increased release of salicylic acid.

In this study, we have developed and tested a series of methods to characterize aerogels and the corresponding release of salicylic acid. Salicylic acid has been shown to have a beneficial effect on plants when administered exogenously [7,8]. However, too high levels of salicylic acid may decrease enzyme activities and inhibit uptake of ions such as potassium [38]. On the contrary, low doses of salicylic acid have been found to be more beneficial, particularly concentrations around 10^−5^ M [39]. The values of salicylic acid released from CNF-aerogels are in this concentration range (Figure 6d), which suggests that the release of salicylic acid may be sufficient. Further work will thus focus on the testing of CNF aerogels loaded with salicylic acid and their effects on plant health.

## 4. Conclusions

We prepared porous structures of CNFs embedded with a plant hormone. A series of methods were implemented and their suitability to characterize the porous structures and the release of salicylic acid was demonstrated. The results of this study indicate that increased surface charge leads to a higher release of salicylic acid, probably due to electrostatic repulsion. The release profile was assessed, and we quantified that roughly 6.5 × 10^−5^ M SA was released after 24 h from the porous CNFs aerogels. We propose that biodegradable constructs of CNFs can be loaded with a plant hormone that is released slowly over time, which suggests uses in small scale agricultural applications and in the private home market.

## Figures and Tables

**Table 1:** Carboxyl group contents of bleached and unbleached cellulose nanofibrils (CNFs).

Sample Name	Carboxyl Content (µmol/g)	Standard Deviation (µmol/g)
Unbleached CNFs	1057	18.4
Bleached CNFs	1376	23.5
